# Reverse Abdominoplasty: A Practical Option for Oncological Trunk Reconstruction

**Published:** 2013-01-14

**Authors:** Nicholas M. Pantelides, Debabrata Mondal, Gordon C. Wishart, Charles M. Malata

**Affiliations:** ^a^Department of Plastic and Reconstructive Surgery; ^b^Cambridge Breast Unit, Addenbrooke's Hospital, Cambridge University Hospitals NHS Foundation Trust; ^c^Faculty of Health and Social Care, Anglia Ruskin University, Cambridge, United Kingdom

## Abstract

**Objectives:** Following radical oncological resection, full-thickness upper central trunk defects present a significant challenge. Common reconstructive options include pedicled flaps, such as pectoralis major, rectus abdominis, and latissimus dorsi. In complex cases, free tissue transfer may be required. Reverse abdominoplasty, although initially described for cosmetic body contouring, can be used to reconstruct upper central trunk defects following radical tumour ablation. We present 4 such applications in the management of advanced or recurrent malignancies and review the relative indications for this approach. **Methods:** Four consecutive cases (2004-2010) were reviewed with respect to indication, operative procedure, and complications. **Results:** There were no cases of complete flap loss. One patient underwent revision for marginal flap necrosis while another developed local recurrence, requiring re-excision and reconstruction with flap advancement. **Conclusions:** Where pedicled flaps are unavailable or insufficient, adjacent abdominal tissue can be recruited into chest wall defects, avoiding microsurgical free tissue transfer. The authors feel that the reverse abdominoplasty is currently underused in this context and offers an excellent alternative in complex cases where other reconstructive options are unavailable, or where comorbidities preclude free-tissue transfer. The technique is versatile, simple to perform and affords an acceptable cosmetic outcome, yet is not widely reported in the literature. It has particular merit in cases with a high chance of disease recurrence, in the management of recurrent breast cancer, and in patients with multiple comorbidities. The reverse abdominoplasty should therefore be considered when evaluating patients for oncological trunk reconstruction.

Central trunk defects following radical ablation of malignancy pose a reconstructive challenge. This is primarily due to the limited availability of local flaps, their reach restrictions, and the uncertainty about achieving clear excision margins. The situation may be further complicated by disease recurrence and the adverse effects of previous radiotherapy on surrounding tissues.

The reverse abdominoplasty flap, first described by Rebello and Franco,^1^ is an advancement flap utilized largely for aesthetic contouring of the upper anterior trunk^2^^-^4 and more recently employed in post–massive weight loss surgery.^5^^-^6 Its use in reconstruction of the chest wall following radical tumor resection is, however, rare.^7^^-^8 We present 4 applications of the reverse abdominoplasty flap for management of the upper central trunk defects following oncological resection.

## METHODS

From 2004 to 2010, 4 reverse abdominoplasty procedures were performed by the senior author for reconstruction of full-thickness upper central trunk defects following oncological resection. The patients, all female, had a mean age of 50 years (range, 32-69) at the time of the initial reverse abdominoplasty procedure. Three cases were performed as elective procedures whereas, in one case, paraneoplastic disease necessitated “emergency” mastectomy and reconstruction. Two patients had received local radiotherapy prior to the procedure.

The average period of follow-up from operation to the time of writing was 47 months. There were no cases of complete flap loss. One patient underwent surgical revision for marginal flap necrosis while another developed local recurrence, which required re-excision and further reconstruction.

### Surgical technique

**Figure 1:**
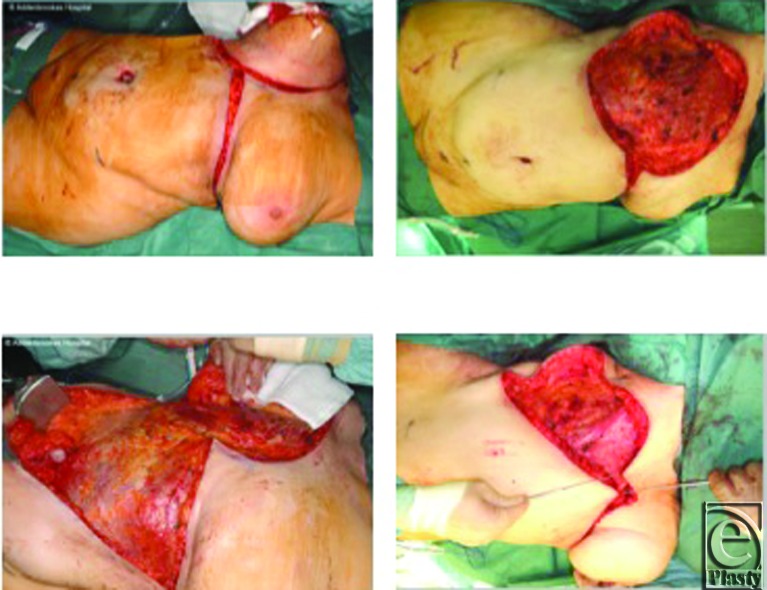
Case 1: An intraoperative sequence. An inframammary “gullwing” incision was made (*top left*) and a large full-thickness defect with exposed pectoralis major muscle was created following radical resection (*top right*). The caudal reverse abdominoplasty flap was undermined to the level of the umbilicus to achieve the necessary reach (*bottom left*) and the flap was advanced cranially to fill the defect (*bottom right*).

Preoperative assessment of abdominal wall laxity is carried out with the patient in both the standing and the recumbent positions. The inferior resection margin of the tumor ablation constitutes the superior border of the abdominoplasty flap and must be adequate and not compromised by concerns to preserve tissue for the reconstruction. The markings may be subsequently revised during surgery according to final size and configuration of the defect present. An inframammary “gull-wing” incision is made, centered on the midline (Fig 1, *top left*). The abdominoplasty flap is raised below Scarpa's fascia, just above the rectus sheath (Fig 1, *bottom left*). The dissection continues beyond the umbilicus to the pubic area and the groin creases on each side. The umbilical stalk is usually divided to allow the umbilicus to “float.” Dissection is aided by the use of a deep or long-lighted retractor or a headlight. Meticulous hemostasis is achieved and 2 suction drains are inserted. The suction drains are placed deep to the advanced flap, carefully avoiding the superficial inferior epigastric vessels. The caudal abdominal tissue is then advanced cranially into the chest wall defect (Fig 1, *bottom right*). The flap is inset using 2/0 PDS sutures from the Scarpa's layer to the superficial fascial system of the chest. Skin closure is performed in layers with 3/0 Monocryl deep dermal, running Monocryl subcuticular followed by Steri-strips (3M, St Paul, Minnesota) applied across the wound.

## CASE REPORT 1

**Figure 2:**
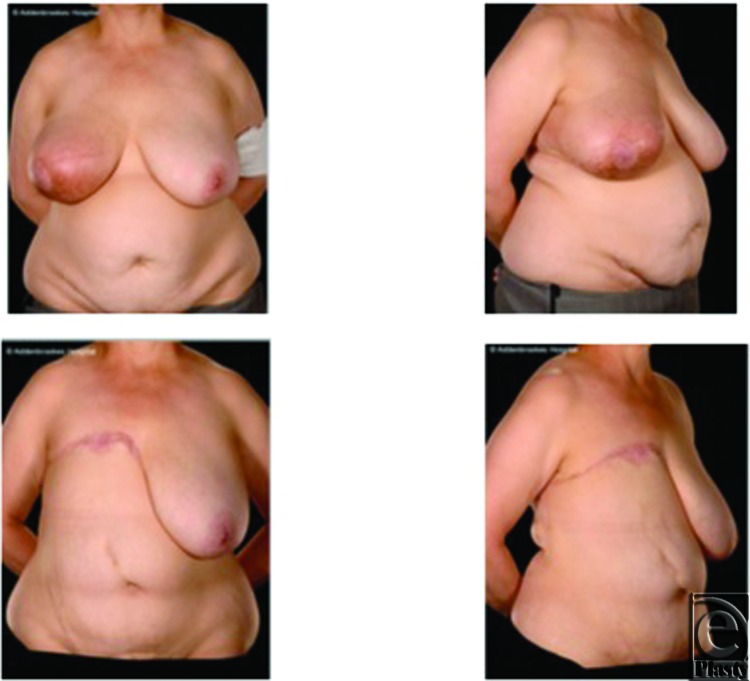
Case 1: Pre- and postoperative appearances. Preoperative appearance showing and inflammatory recurrence in the right breast with visible edema and erythema (*top left* and *right*). Appearances 7 months postoperatively demonstrating a good cosmetic outcome with an acceptable abdominal contour (*bottom left* and *right*).

A 46-year-old woman underwent right wide local excision and axillary clearance following a diagnosis of grade II invasive ductal carcinoma in her right breast. Despite receiving adjuvant chemotherapy and radiotherapy, she re-presented 4 years later with an inflammatory local recurrence (Fig 2, top), which responded poorly to further chemotherapy.

She underwent a right-sided extended mastectomy with a 3-cm circumferential resection margin and excision of the pectoralis fascia. A large full-thickness defect was created, which was reconstructed by advancing a reverse abdominoplasty flap (Fig 1).

The postoperative course was complicated by a small area of fat necrosis in the distal end of the flap, which necessitated debridement and direct (primary) closure. The final postoperative appearances are satisfactory (Fig 2, *bottom*). She remains disease-free and has declined delayed breast reconstruction.

## CASE REPORT 2

A 48-year-old woman presented with a progressively enlarging lump in her upper central abdomen. Her surgical history included an uncomplicated laparoscopic cholecystectomy for gallstones 5 years earlier. Computed tomographic images showed that the mass extended posteriorly to the anterior wall of the stomach and a core biopsy confirmed an adenocarcinoma; the pathological diagnosis was a port-site metastasis originating from a gallbladder tumor. During the next 3 weeks, the tumor enlarged rapidly reaching a palpable 15 × 12 cm epigastric mass with clinically detectable diastasis recti.

**Figure 3:**
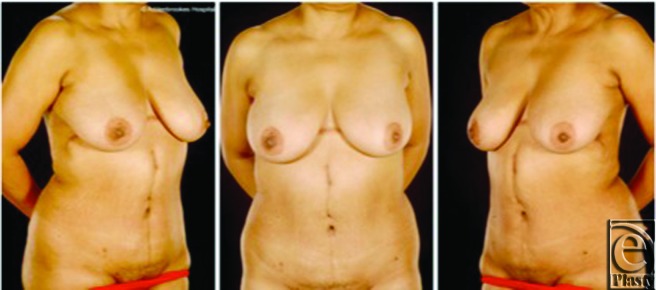
Case 2: Postoperative views 1 year after surgery. This flap affords an acceptable cosmetic appearance with the scar partly concealed by the patient's breasts.

A full-thickness resection of the tumor in the anterior abdominal wall was carried out under frozen section guidance. The lesion was entirely contained within the falciform ligament, but it was necessary to include segments of rectus abdominis muscle in the resection. The resulting defect was reconstructed prosthetically in 2 layers comprising a posterior DynaMesh (FEG Textiltechnik mbH, Aachen, Germany) and an anterior Prolene mesh (Ethicon Inc, Somerville, New Jersey). Skin and soft tissue coverage was achieved with a reverse abdominoplasty advancement flap (Fig 3). There were no postoperative problems and the patient remains clinically and radiologically disease-free 4 years later.

## CASE REPORT 3

A 56-year-old woman underwent breast conserving surgery for primary invasive breast cancer, followed by adjuvant breast radiotherapy. Nine years later, she was diagnosed with radiation-induced angiosarcoma of the same breast, for which she underwent bilateral mastectomy. She remained disease free for the next 4 years, when she developed recurrent angiosarcoma of her right chest wall. The recurrence was resected and the chest wall reconstructed using a right latissimus dorsi myocutaneous flap. An involved superior margin necessitated re-excision and direct closure.

**Figure 4:**
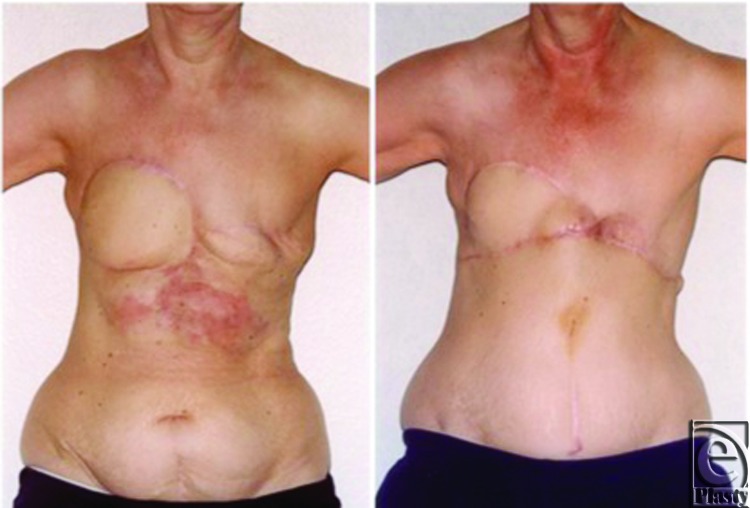
Case 3: Local recurrence of angiosarcoma and reconstruction. Further local recurrence presented as indurated erythema across the upper abdomen measuring 26 × 11 cm inferior to the previous right latissimus dorsi myocutaneous flap reconstruction (*left*). Right photograph illustrates the reconstructive use of the abdominoplasty flap with consequent superior transposition of her umbilicus.

A few months later, she manifested a further local recurrence in the upper abdomen/lower chest wall, which proved resistant to a trial of chemotherapy (Fig 4, *left*). This was resected with a 3-cm margin down to deep fascia, and the defect was reconstructed with a reverse abdominoplasty and full-thickness skin graft (Fig 4, *right*).

**Figure 5:**
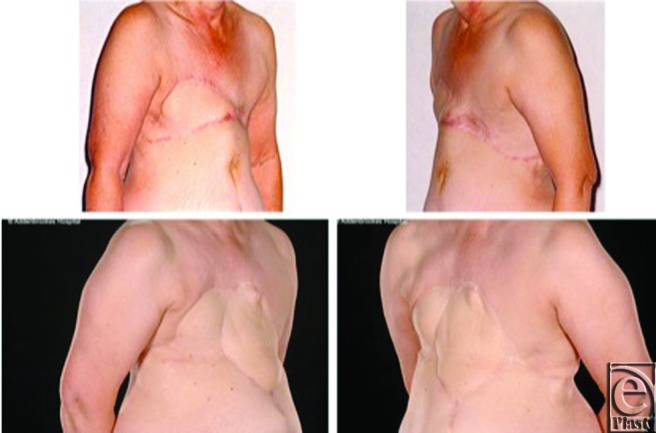
Case 3: Further local recurrence and reconstruction, including advancement of the reverse abdominoplasty. Recurrent disease, visible as a 6 × 8 × 3 cm epigastric bulge above the umbilicus (*top row*), was radically resected and reconstructed with a left latissimus dorsi flap and advancement of the reverse abdominoplasty flap. Appearances 2 years after surgery (*bottom row*) show no local recurrence and an acceptable aesthetic appearance of the 3-flap reconstruction.

She remained disease free for the next 7 months, before re-presenting with rapid disease progression (Fig 5, *top*). This necessitated further resection with at least 3-cm margin, including the xiphisternum, perichondria of the lower 3 ribs and the upper parts of the rectus muscles. There was no peritoneal involvement and the central abdominal wall reconstruction was performed using Prolene mesh (Ethicon Inc, Somerville, New Jersey) and a left latissimus dorsi myocutaneous flap in combination with readvancement of the reverse abdominoplasty flap. Healing was uneventful (Fig 5, *bottom*) and she remained disease free for the next 4 years.

## CASE REPORT 4

A 32-year-old woman presented to the emergency department with a short history of proximal muscle weakness, a bulbar palsy, and a recently enlarging right breast mass. A computed tomographic scan demonstrated an 8 × 8 cm mass in the medial right breast suggestive of malignancy, with right axillary lymphadenopathy but no evidence of distant metastases. Soft tissue biopsies confirmed a paraneoplastic acute necrotizing myopathy and dermatomyositis.

Following a diagnosis of invasive breast cancer, she underwent an urgent right mastectomy and axillary lymph node dissection. This required radical breast skin excision, including the nipple-areolar complex but preserving lateral breast skin. The tumor resection included some pectoralis major muscle for clearance. The resultant defect was closed using a reverse abdominoplasty advancement flap. Histological examination showed a grade III metaplastic carcinoma of the breast with squamous differentiation and extensive lymph node spread.

Postoperatively, she required continued intubation and admission to the intensive care unit. She received intravenous steroids and was discharged home 5 weeks after admission.

An outpatient review 6 weeks following discharge from hospital revealed a 2 × 8 cm area of dehiscence of the lateral mastectomy skin flap, with an 8 × 5 cm cavity deep to this. This has been treated conservatively with negative pressure wound therapy, although there has been a delay in the commencement of adjuvant treatment due to seroma formation and slow healing.

## DISCUSSION

Full-thickness upper central trunk defects following oncological resection present a reconstructive challenge. Local flaps may be unavailable due to involvement of the underlying disease process, whereas flap viability may be compromised by previous or planned chest wall radiotherapy. The potentially recurrent nature of the underlying disease may necessitate wide excision margins followed by multiple ablative and reconstructive procedures. Careful planning is therefore necessary when considering potential and optimal reconstructive options—this may involve local, pedicled or free-flap transfer.

Classification systems^9^^-^11 have been proposed to aid a systematic approach but the exact choice remains very much patient-specific. Rohrich et al^9^ advocate the use of the pedicled superior rectus abdominus flap for medial upper trunk lesions with an extended latissimus dorsi flap as the first-line option for more lateral defects at this level. Other pedicled flap options include pectoralis major and external oblique muscles. These flaps may be used individually or in combination.

More complex reconstructions, often when local tissues have been severely damaged or the pedicled flaps have insufficient size or reach, may require free tissue transfer.^12^ The most commonly used is the tensor fascia lata, often with the superior epigastric, internal thoracic, or a saphenous vein loop graft as the recipient vessel.^13^ In our group, we have used the anterolateral thigh free flap for advanced central chest wall tumors using extrathoracic recipient vessels.^14^ Although free flaps offer more reliable healing when compared to pedicled flaps, owing to their superior blood supply, there are also disadvantages, such as the prolonged operative time and the possibility of total flap failure.

The reverse abdominoplasty offers an alternative to pedicled or free flap reconstruction by recruiting adjacent abdominal tissue into the defect. First described in English by Baroudi et al^2^ in combination with a reduction mammaplasty, it was later popularized by Grazer^3^ as an aesthetic procedure in its own right. Despite its potential as a reconstructive procedure, the vast majority of reverse abdominoplasties are performed for aesthetic purposes.^2^^-^6^,^^15^^-18^ A search of the English-language literature using PubMed reveals only 2 citations since 1984 describing the technique for reconstruction following oncological resection.^7^^,^^8^

Although we do not advocate the reverse abdominoplasty as a first-line choice for oncological trunk reconstruction in all cases, we feel that it is currently underused in this context and offers an excellent alternative in complex cases where other reconstructive options are unavailable, or where comorbidities preclude free-tissue transfer.

The reverse abdominoplasty has many advantages when used for upper trunk reconstruction (Table 1). It can contribute considerable bulk to obliterate defects and can be used in combination with other flaps or skin grafts, or as soft tissue coverage over a prosthetic mesh. Since it requires no preoperative work-up, it is also readily available in the emergency setting, as in case 4. Given its wide base, it is a reliable flap and discontinuous undermining or liposuction are not required to preserve vascularity. It provides local tissue with similar texture and appearance and the resulting cosmetic appearance is acceptable, with the scar partly concealed by the breasts, as evident in the postoperative images of case 2 (Fig 3).

The reverse abdominoplasty has particular merit in cases of locally advanced disease where the risk of recurrence is high, or in recurrent breast cancer. Where further resection is necessary, it is much simpler to remove part of the reverse abdominoplasty flap and advance the tissues than to undertake resection of a pedicled or free flap, as is commonly performed. If there is insufficient tissue, the reverse abdominoplasty can be combined easily with other flaps, and the option of free tissue transfer remains available as a last resort. In the case of recurrent breast cancer, the use of the reverse abdominoplasty spares certain potential reconstructive options, such as a pedicled latissimus dorsi flap, which can be considered for secondary reconstruction after a suitable disease-free interval. However, given the advanced and recurrent nature of the cases we describe, none of our patients sought consultation for secondary breast reconstruction.

Using this relatively simple technique, we were able to avoid microsurgical free tissue transfer despite the complex nature of primary and recurrent disease described in our case series. This was possible partly because the patients were parous women, who thus had adequate lower abdominal laxity, which could be mobilized to enable abdominoplasty flap advancement superiorly.

## CONCLUSION

Following radical oncological resection, full-thickness upper central trunk defects pose a reconstructive challenge. The authors believe that in selected patients, successful reconstruction of central trunk defects following radical tumor ablation can be achieved using the reverse abdominoplasty, without resorting to microsurgical free tissue transfer. We therefore advocate consideration of this technique when evaluating patients for upper central trunk defect reconstruction.

## Figures and Tables

**Table 1:** Advantages and disadvantages of the reverse abdominoplasty flap

Advantages/Indications
• Reliable flap vascularity
• Ease of dissection
• Contributes significant bulk
• Can be combined with other flaps, grafts, or meshes as necessary
• Available in the emergency setting
• Can be partially resected or readvanced if required
• Good cosmetic outcome
• No muscle dissection so minimal functional sequelae
• Avoids microsurgical free tissue transfer where this is undesirable
Disadvantages/Limitations
• Less reliable healing at the leading end when compared to free tissue transfer
• Extensive undermining precludes future transverse rectus abdominis myocutaneous/deep inferior epigastric perforator reconstruction
• May be unreliable in the obese
